# The mental health and well-being profile of young adults using social media

**DOI:** 10.1038/s44184-022-00011-w

**Published:** 2022-09-07

**Authors:** Nina H. Di Cara, Lizzy Winstone, Luke Sloan, Oliver S. P. Davis, Claire M. A. Haworth

**Affiliations:** 1 Department of Population Health Science, University of Bristol, Bristol, UK; 2 MRC Integrative Epidemiology Unit, University of Bristol, Bristol, UK; 3 Cardiff University, Cardiff, Wales, UK; 4 The Alan Turing Institute, London, UK; 5 Department of Psychological Science, University of Bristol, Bristol, UK

## Abstract

The relationship between mental health and social media has received significant research and policy attention. However, there is little population-representative data about who social media users are which limits understanding of confounding factors between mental health and social media. Here we profile users of Facebook, Twitter, Instagram, Snapchat and YouTube from the Avon Longitudinal Study of Parents and Children population cohort (*N* = 4083). We provide estimates of demographics and mental health and well-being outcomes by platform. We find that users of different platforms and frequencies are not homogeneous. User groups differ primarily by sex and YouTube users are the most likely to have poorer mental health outcomes. Instagram and Snapchat users tend to have higher well-being than the other social media sites considered. Relationships between use-frequency and well-being differ depending on the specific well-being construct measured. The reproducibility of future research may be improved by stratifying by sex and being specific about the well-being constructs used.

## Introduction

The trails of data left online by our digital footprints are increasingly being used to measure and understand our health and well-being. Data sourced from social media platforms has been of particular interest given their potential to be used as a form of ‘natural’ observational data about anything from our voting intentions to symptoms of disease. There is not a single, widely agreed definition of the term ‘social media’^1^, but for the purposes of this study we understand it to be a broad category of internet-based platforms that allow for the exchange of user-generated content by ‘users’ of that platform^2^. Both the huge volumes of data available on such platforms, and their increasing uptake across the population^3^ have led to two main fields of interest in the intersections of social media and mental health. These are the prediction of mental health and well-being from our online data^4^ and, somewhat reciprocally, the influence of social media on our mental health, particularly in the case of children and young people^5,6^. These fields both ask fundamental questions about the mental health and well-being of social media users, to either understand the ways our mental health influences our social media behaviour, or how our social media behaviours influence our mental health.

Across both contexts a wide range of psychological outcomes have been studied, including predicting suicide at a population-level^7^ and individually^8^, mapping the influences of social media platforms on disordered eating^9^ and self-harm^10^, understanding the impacts of cyberbullying through social media platforms^11,12^, and even ethnographic research into online support networks^13^. As highlighted in a recent review which considered research on the relationship between social media use and well-being in adolescents^14^, there has tended to be an inherent assumption that social media is the cause of harm when examining the effect of social media on our health. However, recent investigations such as those by Orben and Przybylski^15,16^ and Appel and colleagues^17^ illustrate that the role of social media in causing harm may be over-estimated. It seems likely that there is some reciprocal relationship between mental health and social media, that requires longitudinal research studies to begin to understand the complexity, coupled with large representative samples to explore the heterogeneity^18,19^. Further, there is increasing attention on the role of within-person effects that see impact change between contexts^20,21^, as well as individual differences^22^. Meanwhile, attention has also been drawn to the comparative lack of investigation into the potential benefits of social media, such as access to peer support and the ability to readily connect with friends and family, or into the psychological well-being of social media users as opposed to focusing on pathology. Similarly, most psychological prediction tasks using social media focus on predicting illness rather than wellness^4,23^.

Regardless of the direction of interest in the relationship between social media and psychological outcomes, researchers face common challenges, with one of the primary issues being a lack of high-quality information on the characteristics of the whole population of social media users^24^. Valuable demographic information on social media users in the United States is regularly produced by the Pew Research Centre^25^, but often researchers rely on algorithmic means to make predictions about the demographics of the groups they study online if they are not recruiting a participant sample whose demographics are known and can be recorded^4,24,26^. What we do know about social media users is that they are not homogeneous. The demographic features of populations using them vary across platforms and do not tend to be consistent with the characteristics of the general population^25–28^. This work on the demographic context has been important in understanding the samples that can be drawn from social media platforms, but there remains a lack of information about other characteristics of social media users that are relevant to study outcomes, including mental health and well-being. Consequently, attempts to compare user well-being and mental health between platforms may be unknowingly confounded by differences in the mental health profile of each individual platform. Mellon and Prosser^28^ investigated this form of selection bias with respect to differences in political opinion between Facebook and Twitter, and noted the potential for study outcomes to be biased when the outcome variable of interest is associated with the probability of being included in the sample^29^. This also has implications for our assessment of mental health and well-being classification algorithms^30^. For instance, if using Twitter data to classify depression in a random sample of users how many of these users should we expect to be depressed? Should we expect to find more depressed users on Facebook or Instagram? This bench-marking would allow the research community, who frequently face the challenge of establishing reliable ground truth in social media research, to contextualise the sensitivity and specificity of developed models^4,24^.

This study aimed to address the gap in the availability of high-quality descriptive data about social media users by describing social media use in a representative UK population cohort study, the Avon Longitudinal Study of Parents and Children (ALSPAC)^31^. We aimed to profile the users of the social media platforms Facebook, Instagram, Twitter, Snapchat and YouTube by considering a range of mental health and well-being measures that are regularly studied, with the objective of better characterising social media users against variables of interest to researchers. These measures included disordered eating, self-harm, suicidal thoughts, and depression as well as positive well-being outcomes which are sometimes neglected in the context of social media research^14,16,22^ like subjective happiness, mental well-being and fulfilment of basic psychological needs. In answering our research questions we also sought to illustrate how cross-sectional data from a representative population cohort *can* provide meaningful contextual information that informs the way we interpret past and future research about social media users and their mental health. Unlike other studies using cross-sectional data^14^ we had no intention of exploring causal questions, but aimed to address unanswered questions of who social media users are, and whether selection bias across platforms may have the potential to unintentionally bias outcome statistics about mental health and well-being.

Specifically, our research questions were:

(1) Are there demographic differences in patterns of social media use (e.g. frequency)?
(2) Are there demographic differences in the user groups of different social media platforms?
(3) Are there differences in the mental health and well-being of those using social media sites at different frequencies?
(4) Are there differences in the mental health and well-being of user groups of different social media platforms?

## Methods

### Sample description

The sample for this study is drawn from the Avon Longitudinal Study of Parents and Children (ALSPAC)^31–33^. Pregnant women resident in Avon, UK with expected dates of delivery from 1st April 1991 to 31st December 1992 were invited to take part in the study. The initial number of pregnancies enrolled was 14,541. Of these initial pregnancies, 13,988 children were alive at 1 year of age. When the oldest children were ~7 years of age an additional 913 children were enrolled. The total sample size for ALSPAC of children alive at one year of age is 14,901. However, since this time there has been a reduction in the sample due to withdrawals, deaths of those in the cohort and also people simply being lost to follow-up. As such the exact number of participants invited to each data collection activity changes with time. Please note that the ALSPAC study website contains details of all the data that is available through a data dictionary and variable search tool (http://www.bristol.ac.uk/alspac/researchers/our-data/). Study data were collected and managed using REDCap electronic data capture tools hosted at the University of Bristol^34^.

The analysis presented in this study is based on a sub-sample of 4083 participants who responded to a self-report questionnaire at a mean age of 24 years old in 2016/17. The survey was sent to 9211 currently enrolled and contactable participants, of whom 4345 (47%) returned it. To maintain a consistent sample throughout the following analyses we considered the 4083 observations with complete cases for questions related to self-harm, suicidal thoughts, disordered eating, and social media use, and without the respondents who said that they ‘didn’t know’ whether they had a social media account (*n* < 5); no respondents stated that they did not have a social media account. As well as the survey at age 24, we considered the responses by those in our main sample to a survey one year previously, at age 23, which collected the well-being measures and the Moods and Feelings Questionnaire, matched to their social media use responses at age 24. This resulted in a sub-sample of 2991 participants who had responded to both surveys. Table 1 gives a comparison of the demographic breakdowns across these samples.

### Measures

This study considered the participants’ responses to a range of mental health and well-being measures, as well as demographic data. A brief overview of each of the measures used is given below.

Throughout this paper, we used *Male* and *Female* to refer to the participant’s assigned sex at birth. Participant ethnicity was reported by their parent/s, and is available in the data as *White, Ethnic Minority Group*, or *Unknown*, where Ethnic Minority Group was only available as one group rather than broken down into specific ethnicities. There were two variables relevant to socio-economic status. The first was whether the participant had achieved an A Level or equivalent qualification by age 20, the second was their parents’ occupation. Parental occupation was measured using the Registrar General’s Social Class schema^35^, and was collected prior to the birth of the index cohort; we took the higher occupational class of the participant’s parents where available and grouped the overall schema of six categories into those in *manual work*, and those in *non-manual work*.

Social media use was measured using three questions. These were: (1) *Do you have a social media profile or account on any sites or apps?* with possible responses of ‘Yes’, ‘No’ or ‘Don’t know’; (2) Given a list of social media sites, *Do you have a page or profile on these sites or apps, and how often do you use them?*, where the social media sites were listed and response options were ‘Daily’, ‘Weekly’, ‘Monthly’, ‘Less Than Monthly’ or ‘Never’; (3) *How often do you visit any social media sites or apps, using any device?* with response options being ‘More than 10 times per day’, ‘2 to 10 times per day’, ‘Once per day’ or ‘Less than once per day’. Here, the definition of ‘social media sites’ in questions (1) and (3) was left to the participant to interpret, whereas in (2) a specific list was provided. In the following analyses, we have summed responses for the use frequencies per platform from question (2) so that ‘Weekly’, ‘Monthly’ and ‘Less than monthly’ are combined to represent ‘Less than daily’.

Depressive symptoms were measured using the short Mood and Feelings Questionnaire (MFQ)^36^, a 13-item scale that has been validated for measuring depressive symptoms in adolescents^37^ and in young adulthood^38^. It asks respondents to rate statements, such as *I cried a lot* and *I thought nobody really loved me*, as *Not true, Sometimes* or *True* based on how they felt over the past two weeks. Missing items were filled with the mode of the individual’s other responses, provided 50% or more of the items were completed. Scores range from 0 to 26, with a higher score indicating more severe depressive symptoms^37^. Here we applied a cut-off score of 12 or above as indicating depression^38^.

Suicidal thoughts were assessed with the question *Have you ever thought of killing yourself, even if you would not really do it?* with those who indicated that they had ‘within the past year’ being included. Similarly, intentional self-harm was assessed by asking if participants had *hurt [themselves] on purpose in any way* and we included those who said this had happened at least once within the last year.

Disordered eating was a composite variable that included participants who indicated that they had been told by a healthcare professional that they had an eating disorder (anorexia nervosa, bulimia nervosa, binge eating disorder or another unspecified eating disorder). Participants were also included if they indicated they had engaged in any of the following behaviours at least once a month over the past year with the intention of losing weight or avoiding weight gain: fasting, throwing up, taking laxatives or medication. This classification of disordered eating followed a similar methodology to that used by Micali and colleagues^39^.

Well-being was measured using seven questionnaires. The Warwick Edinburgh Mental Well-being Scale (WEMWBS) is a fourteen-item questionnaire that has been validated for measuring general well-being in the general population^40,41^, as well as in young people^42,43^. It asks respondents to rate statements such as *I’ve been dealing with problems well* and *I’ve been feeling cheerful*, on a five-point Likert-type scale. The total score is between 14 and 70. All items in the WEMWBS are positively worded, and it is focused on measuring positive mental health.

The Satisfaction with Life Scale^44,45^ is five-item questionnaire designed to measure global cognitive judgements of satisfaction with one’s life, which includes statements such as *If I could live my life over, I would change almost nothing*. Each question uses a seven-point Likert-type measure and the total score is between 5 and 35. The Subjective Happiness Scale^46^ is a four-item questionnaire based on seven-point Likert-type questions, with the overall score being a mean of the four questions, lying in the range of 1 to 7. Respondents answer questions such as whether they consider themselves to be more or less happy than their peers.

The Gratitude Questionnaire (GQ-6) is a six-item measure that uses a seven-point Likert-type scale to assess individual differences in proneness to experiencing gratitude in daily life^47^. This scale includes statements such as *I have so much in life to be thankful for* and *I am grateful to a wide variety of people*. Each score is summed to a total between 6 and 42. The Life Orientation Test (LOT-R) is a measure of dispositional optimism that has ten items asked on a 5-point Likert-type scale^48^, though only four of these items are ‘filler’ questions that do not contribute to the final score. The overall score is in the range of 0 to 24, and items that contribute to this include *In uncertain times, I usually expect the best* and *I hardly ever expect things to go my way*.

The Meaning in Life questionnaire has 10 items designed to measure two dimensions of meaning in life: (1) Presence of Meaning (how much respondents feel their lives have meaning), and (2) Search for Meaning (how much respondents strive to find meaning and understanding in their lives)^49^. Statements include *I understand my life’s meaning* in the Presence sub-scale, and *I am looking for something that makes my life feel meaningful* in the Search sub-scale. Respondents answered each item on a 7-point Likert-type scale, with the two sub-scales scored in total between 5 and 35.

The psychological constructs of autonomy, competence and relatedness associated with self-determination theory were measured using the Basic Psychological Needs in General (BPN) questionnaire^50^. This questionnaire has 21 seven-point Likert-style questions with the final score for each of the three sub-domains being the mean of the responses for that sub-domain. As such each of autonomy, competence and relatedness were scored overall from 1 to 7. Example items include *People in my life care about me* and *I often do not feel very capable*.

For all measures missing items were filled with the person-level average, provided that half or more of the items were completed. All of the well-being measures listed were scored in a positive direction, where higher scores indicate higher alignment with the construct being measured.

### Analysis

The descriptive statistics were calculated using the R programming language (v4.0.1)^51^ in RStudio (v1.3), primarily using the tidyverse (v1.3.0) package^52^ for data manipulation and ggplot2 (v3.3.1)^53^ for visualisation. A reproducible version of the manuscript and supporting code can be found from the Code availability statement.

### Ethics

Ethical approval for the study was obtained from the ALSPAC Ethics and Law Committee and the Local Research Ethics Committees. Informed consent for the use of data collected via questionnaires and clinics was obtained from participants following the recommendations of the ALSPAC Ethics and Law Committee at the time. The full list of ethical approval references for ALSPAC can be found on their website (https://www.bristol.ac.uk/alspac/researchers/research-ethics/).

## Results

### Demographics

We first consider the demographics of social media users across different frequencies of use, and across the five social media platforms: Facebook, Twitter, Instagram, Snapchat and YouTube. These are both taken from the main sample, as described in our ‘Methods’. Table 2 presents the frequency that participants reported using any social media sites each day, based on sex, ethnicity, education, and their parents’ occupational group.

Table 3 gives the percentage of participants from each demographic group who reported being a user of each platform with any use frequency.

**Fig. 1:**
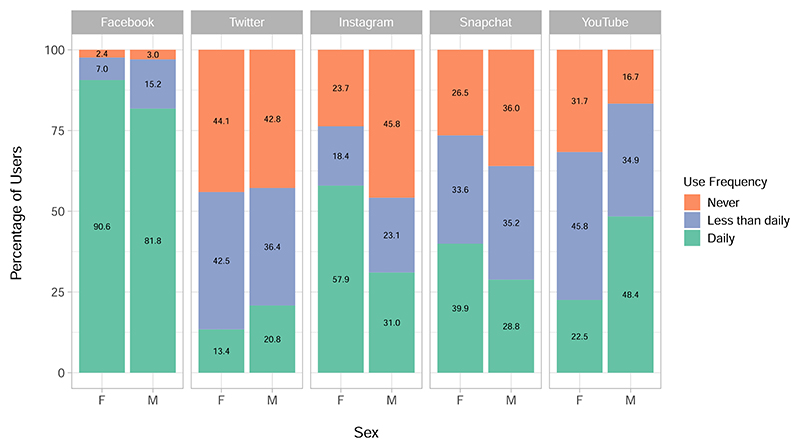
Percentage of participants using each of Facebook, Twitter, Instagram, Snapchat and YouTube by use frequency and sex. All social media users in the sample (*N* = 4083) are split by female (*N* = 2698) and male (*N* = 1385), and the frequency with which they use each social media platform given as either ‘Daily’, ‘Less than daily’ or ‘Never’. Labels on the stacked charts give the precise percentage of the group in each of the frequencies for each platform.

The breakdown of every demographic by frequency of use on each platform is provided in full in Supplementary Table 1. Figure 1 illustrates this breakdown for sex, which is the demographic by which all our following results are stratified due to the imbalance in our sample and the results in Tables 2 and 3. Social media use and mental health and well-being outcomes are also known to vary according to gender^54–56^.

### Mental health and well-being

**Fig. 2:**
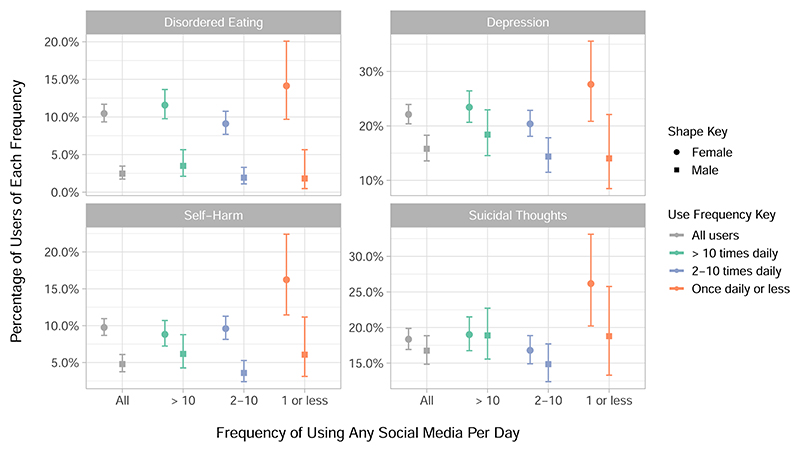
Percentage of participants who reported disordered eating, self-harm or suicidal thoughts in the past year, by sex and frequency of using any social media. The frequency with which participants used any social media is reported as ‘more than ten times a day’, ‘between two and ten times a day’ or ‘once or less per day’, and the percentage of participants in that group who reported each mental health outcome is given in each sub-plot, with 95% confidence intervals. Disordered eating, self-harm and suicidal thoughts were assessed in the main sample alongside the social media questions (*N* = 4083) and included for those participants who reported them in the past year. Depression (*N* = 2991) was measured in the sub-sample with the Moods and Feelings questionnaire in the year prior to the social media measurement, and uses a cut off of 12 or more to indicate the presence of depression.

First we will consider well-being and indicators of poor mental health across different use frequencies. Figure 2 shows how indicators of poor mental health vary across the three frequencies of use, which are more than 10 times a day, 2–10 times a day and once per day or less; no participants reported using no social media at all. These frequencies are contextualised by the prevalence of each outcome in all users of social media. This figure shows that the lowest category of social media use, that is once per day or less, has the highest proportions of disordered eating, self-harm and suicidal thoughts among women. As seen in Table 2, only 7.1% of women and 12% of men used social media less than once per day, and so these measurements are subject to wider confidence intervals. Here, depression is defined as being present in those who scored above the cut-off score of 12 in the Short Mood and Feelings Questionnaire (MFQ)^38^. Additional descriptive data about mental health outcomes in the sample is also available in Supplementary Figure 1 and in Supplementary Tables 2 to 6.

**Fig. 3:**
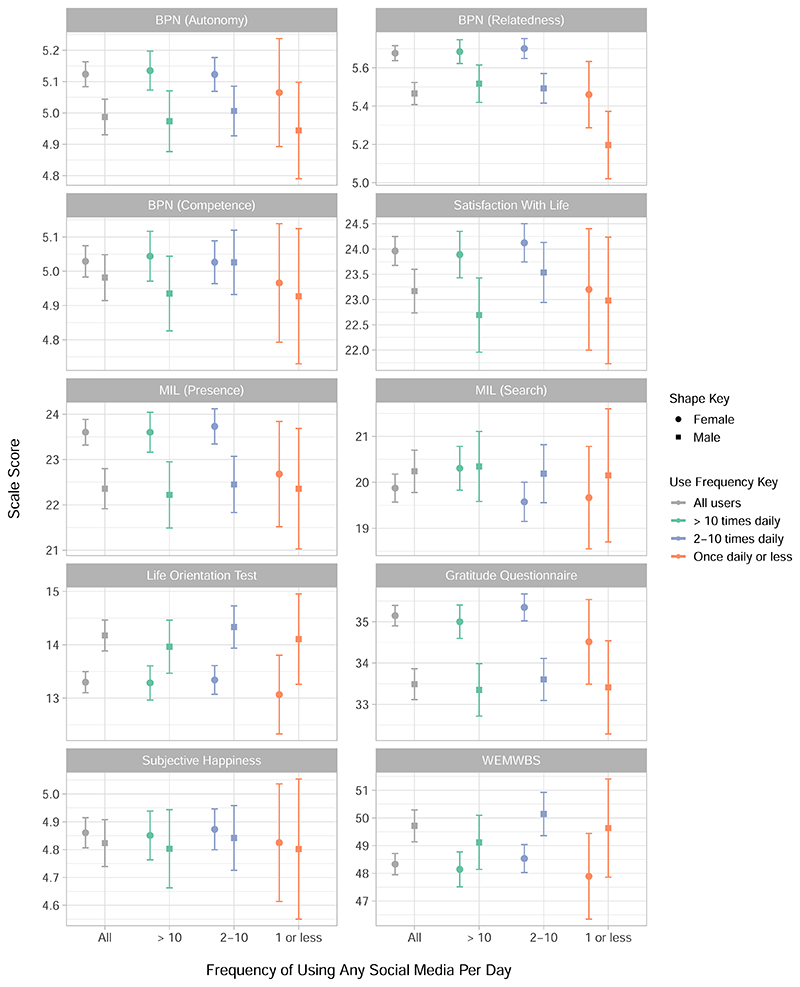
Mean scores for seven well-being measures, by sex and frequency of using any social media. Each sub-graph presents each of the seven well-being measures, including the Basic Psychological Needs scale (BPN) sub-scales autonomy, relatedness and competence, and the Meaning In Life (MIL) scale’s two sub-scales of presence and search. Satisfaction With Life, the Life Orientation Test, the Gratitude Questionnaire, Subjective Happiness Scale and the Warwick Edinburgh Mental Wellbeing Scale (WEMWBS) are also included. The mean of each scale is given for all participants (*N* = 2991) with 95% confidence intervals, split by male and female, and then for each dichotomous category of use-frequency which is one of ‘more than ten times a day’, ‘between two and ten times a day’ or ‘once or less per day’.

Similarly, each well-being construct is presented in Fig. 3, and contextualised by the result for all users of social media, regardless of frequency. Separate outcomes are presented for the three sub-scales of the Basic Psychological Needs (BPN) scale and the two sub-scales of the Meaning in Life (MIL) scale. The Life Orientation Test measures optimism, and the Warwick Edinburgh Mental Well-being Scale (WEMWBS) measures overall positive well-being.

**Fig. 4:**
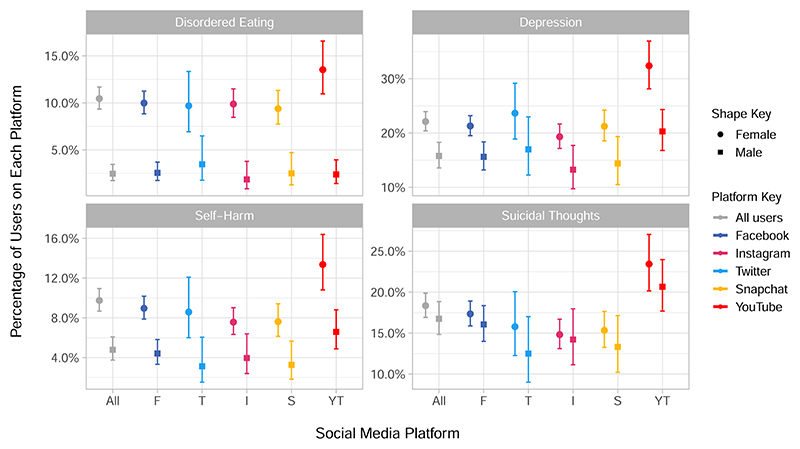
Percentage of participants who reported disordered eating, self-harm or suicidal thoughts in the past year, or who met the threshold for depression, by sex and daily users of each platform. The percentage of daily users of each platform who have reported each symptom is given in each sub-graph, with 95% confidence intervals. Disordered eating, self-harm and suicidal thoughts were assessed in the main sample alongside the social media questions (*N* = 4083) and included for those participants who reported them in the past year. Depression (*N* = 2991) was measured in the sub-sample with the Moods and Feelings questionnaire in the year prior to the social media measurement, and uses a cut off of 12 or more to indicate the presence of depression. Participants can belong to the daily user group of more than one platform.

**Fig. 5:**
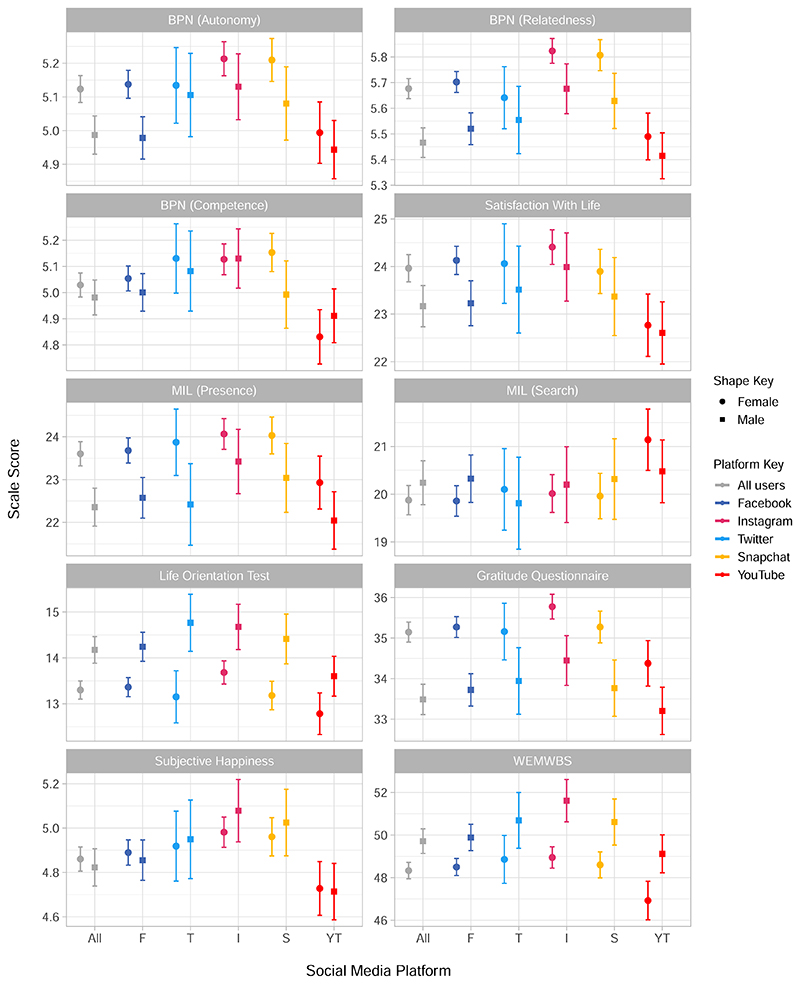
Mean scores for seven well-being measures for daily users of each platform by sex. Each sub-graph presents each of the seven well-being measures, including the Basic Psychological Needs scale (BPN) sub-scales autonomy, relatedness and competence, and the Meaning In Life (MIL) scale’s two sub-scales of presence and search. Satisfaction With Life, the Life Orientation Test, the Gratitude Questionnaire, Subjective Happiness Scale and the Warwick Edinburgh Mental Wellbeing Scale (WEMWBS) are also included. The mean of each scale is given for all daily users of each platform from the sub-sample (*N* = 2991) with 95% confidence intervals, split by male and female.

Next we consider the characteristics of daily users of each platform. The relative percentage of daily users against other types of users for each platform can be referred to in Fig. 1, and versions of Figs. 4 and 5 for all users of each platform are given in Supplementary Figures 2 and 3.

Finally Fig. 5 gives the mean well-being score across each platform for each of the seven well-being measures.

## Discussion

This study used data from a UK population cohort study to describe the demographics and key mental health and well-being indicators of social media users by their self-reported frequency of using social media and five different platforms used at ages 23 and 24. Overall, we saw that there were differences in demographics and mental states of users across use-patterns and platforms used. In the following sections, we detail and discuss the implications of these findings for future research across the themes of demographics, use-frequency and platform used.

In general, just over half of participants reported using social media 2–10 times per day, with more than ten times per day still being common at 39%, and only approximately one in ten participants using social media once per day or less. The results showed that those who rated their social media use at the highest frequency (more than ten times per day) were more likely to be women, more likely to be White and more likely have parents who worked in manual occupations. However, sex was the only demographic that appeared to have a statistical relationship with frequency of use, based on a Chi-squared test. Davies and colleagues^57^ saw similar results from a Welsh population survey of social media use that found there was a difference in social media use across genders, but not by measures of deprivation.

Figure 1 showed that Facebook is, unsurprisingly, the most popular platform both in being used by 97% of the participants and being the most used platform on a daily basis. Instagram and YouTube showed substantial differences in use patterns across male and female users, with approximately double the percentage of women using Instagram daily as men and, conversely, approximately double the percentage of men using YouTube daily as women. Snapchat also saw higher proportions of daily and overall female users, though this difference between sexes was not as dramatic as for Instagram and YouTube. These patterns of use generally agree with the demographics of users on these sites reported for 18–29-year-old US adults by the Pew Research Center^25^, although our sample saw slightly more Twitter users than their estimated 38%, and fewer YouTube users than their estimated 91% (see Table 3). This difference in YouTube users may be partly explained by the fact that it is the only platform with a substantially higher proportion of men than women using it (68% of women vs 83% of men), and that men were under represented in our sample overall compared to women. This emphasises the importance of stratifying results by sex.

Previous research into the demographics of UK Twitter users also aligns with our findings that men and people from higher socio-economic backgrounds are more likely to be Twitter users than women^26,28^. Here, we also saw that those from ethnic minority groups are more likely to be Twitter users than White participants, though this is limited by the fact that we could not further separate out results for people with different ethnicities due to the variables available. Across our sample, Twitter was the only social media platform that had a noticeably higher proportion of both A Level educated participants and parents in non-manual occupations. Snapchat saw the reverse pattern with a higher proportion of participants who did not have A Level qualifications and a higher proportion of participants whose parents worked in manual occupations.

Overall, the sex differences between all male and female users varied across outcomes. For instance, a higher percentage of women experienced depression, disordered eating and self-harm overall, but the gap in the prevalence of suicidal thoughts between men and women was much smaller. This concurs with evidence from the last UK-wide psychiatric morbidity survey, in that ‘common mental health disorders’ are more prevalent in women than men^58^. When it came to well-being, we saw that women also displayed higher mean levels of well-being across most measures. Exceptions were the Life Orientation Test, which showed men generally had higher levels of optimism, the Subjective Happiness Scale where scores were roughly equivalent, and the WEMWBS where men’s general well-being was slightly higher. These results, apart from the WEMWBS, are consistent with findings on UK-wide well-being at the time of the survey, and that men tend to have higher optimism in general^59,60^. Previous research into the WEMWBS has not generally found large sex differences, but there is evidence that in younger samples there are differences that may be explained by socio-economic status^40,41,61^; we note that higher attrition of men in our sample was likely to lead to a bias towards men who are more socio-economically privileged, which may explain why they had higher well-being.

The patterns of mental health outcomes by use frequency displayed in Fig. 2 showed some support for the so-called ‘Goldilocks theory’ of social media use that hypothesises a quadratic, rather than linear, stimulus-response relationship between social media use and mental well-being^62^. This would mean that moderate use of social media, rather than very little or excessive use, is best for well-being. However, this pattern did not consistently apply. For instance, there was an inverse relationship between social media use and percentage of women who self-harm, and in men only the group with the highest level of social media use had more severe depressive symptoms. Previous research has found that in young women higher social media use was associated with increased risk of self-harm^63^, which is in contrast to our results. Similarly, research using the Millennium Cohort Study also found an increasing relationship between objectively measured number of hours spent on social media and how many respondents had clinically relevant symptoms of depression^64^, with a greater increase for girls than boys. Our findings roughly concur with those for the boys, but in women we found that those who used social media the least had the highest rates of depression. However, these differences in findings could reflect the difference in the age of participants or the ways that social media was measured differently across studies. Here we were using use-frequency as categorised into three groups which, as we discuss further in our limitations, may be more reflective of the individual’s mental health and relationship with social media than how frequently they use it^65^.

When considering the results by well-being measure in Fig. 3 we saw that subjective happiness and optimism as measured by the Life Orientation Test both appeared relatively consistent across use categories. Relatedness presented the clearest difference across use categories, with relatedness in women being higher for the two most frequent use frequencies. However, perhaps the most notable outcome was the inconsistency between well-being scales which implies that the choice of scale could affect the interpretation of the impact of well-being on social media use. Research into the relationship between social media use and well-being has been said to suffer from what is known as the ‘jingle-jangle’ paradox where the term ‘well-being’ is used as a catch-all for anything from depression rates to life satisfaction^66,67^. This conflation of different well-being measures leads to comparisons of different psychological constructs which may interact differently with social media use: this is hypothesised as one of the reasons that researchers find conflicting evidence for this relationship^66^, which our results support. This also adds to the picture of researcher degrees of freedom in choosing how to measure psychological constructs, which has been shown to have a substantial impact on the outcome of analyses of social media and mental health^15^. Subjective well-being is a complex and multi-faceted psychological concept^68,69^, and these findings illustrate the importance of recognising that different measures of well-being could imply different relationships between social media and “well-being”.

When considering participant outcomes by daily users of each platform more consistent patterns emerge than for use-frequencies. We saw that, particularly for women, YouTube had the highest proportion of users reporting disordered eating, self-harm, suicidal thoughts and depression, with higher prevalence of depression in female users of YouTube compared to male users (Fig. 4). Whilst overall mental well-being across platforms, as measured by the WEMWBS in Fig. 5, shows YouTube as being marginally but not drastically lower than other platforms, other well-being measures illustrated some key differences. For instance, YouTube users had lower life satisfaction, relatedness and, particularly for female users, levels of competence (Fig. 5). Conversely, daily users of Instagram, and in some cases Snapchat, appeared to have the highest subjective well-being across most measures, with this being particularly noticeable for relatedness, gratitude and happiness (Fig. 5). The role of self-determination theory in social media use has previously been explored for Facebook and social media in general^70^ with relatedness hypothesised as a key motivating factor for social media use. Previous findings have shown that Instagram and Snapchat are used more for social interaction than Twitter and Facebook^71^, and so our results may corroborate the importance of relatedness in the use of particular platforms. Regardless of the specific measure, our results have illustrated that there is variation amongst platforms which further challenges the idea that ‘social media’ or ‘social networking sites’ are a homogeneous group, and reiterates the importance of understanding the context of research about or using social media^28,71^.

At face value, our results appear to directly contrast with the outcomes of the *Status of Mind* report published by the Royal Society for Public Health^72^, where young people rated YouTube as being the most beneficial site for their well-being and Instagram as the worst, based on health-related outcomes such as their anxiety and depression. Our findings that a higher prevalence of YouTube users suffer from poorer mental health and well-being may mean that whilst some platforms are seen as ‘worse’ for young people’s mental health, that does not equate to finding more unwell young people on those platforms. One explanation may be that those experiencing poorer mental health are more likely to use YouTube because they experience more benefits to their mental health from YouTube, such as community building and peer support^13^, than they do from spending time on sites like Instagram. However, this is certainly an interesting area for further exploration in future quantitative and qualitative research.

Whilst this research draws evidence from a robust and well-documented study and the sample being from a birth cohort means that our results are not confounded by age, there are limitations to the cohort sample that we have used. Firstly, the cohort measures a specific age group so we can only infer information about a single age group at each measurement time point. We suspect that different patterns might be found at different ages, knowing that rates of various mental health conditions such as anxiety, depression and suicidality change over the course of childhood, adolescence and adulthood^73^, and since each generation may use social media differently^74^. It is also important to note that the two data collection points used in this study were taken a year apart, and so not all measures were taken exactly at the same time. This means that although we have primarily considered the data cross-sectionally there is a potential for some longitudinal effects to have influenced the data. Secondly, as discussed in the ‘Methods’ section, there was also a limitation in that ethnicity was only available as two categories (White or Ethnic Minority Groups) and so it was not possible to look further into differences in social media by users of difference ethnicities. Additionally, the make up of the area of Bristol that ALSPAC represents is predominantly White. Given these limitations of the sample it would be valuable to conduct similar research in other cohorts that represent more diverse areas. Thirdly, ALSPAC has seen differential attrition over time and so, as seen in Table 1, the sample for this study when the index cohort were in their early twenties has fewer men than women, and more participants from privileged socio-economic groups in terms of education and class background^31^. As well as this, typical social media use changes over time and by age^25^, and so further assessment of social media use across a variety of population-representative age groups would be the most effective way to understand differences between generations.

Another limitation of this study is a lack of specificity about the nature of social media use that participants are referring to when responding. It is possible that activities related to ‘using’ social media, such as posting content versus passive use, change depending on platform used and that there are individual preferences to account for^54,71,75,76^. For instance, YouTube is distinct from other platforms in this study in that its primary function is passive content consumption as opposed to social networking. Previous research has suggested a reciprocal association between passive social media use and lower subjective well-being^75^, whilst using social media for direct communication has been positively associated with perceived friend support^77^. This may better reflect the uses of platforms like Snapchat. As well as the subjective nature of ‘use’, there are also ongoing concerns about using self-reported measures of use-frequency to measure social media behaviours^78–80^. Emerging evidence is showing that self-reports do not align well with objective measurement due to recall bias and differences in interpreting how to include notifications or fleeting checks of social media^79,80^ with self-reported smartphone pickups underestimating associations with mental health compared to objective measures of use^65^. It might be that different ways of measuring social media use, such as types of use, are more useful when considering associations with mental health and well-being outcomes^54^. It is worth noting that the use-frequency measures used in this study are distinct from screen-time, and equivalent use-frequency across platforms may have different time implications; someone may spend short amounts of time on Instagram or Snapchat checking notifications, but do so frequently, versus visiting YouTube once in a day but spending several hours watching content. These nuances are challenging to capture, but by reporting on mental health prevalence across the available responses in a cohort study we can add to the growing understanding of how self-reported social media use frequency is related to mental health. Statistical modelling to test the extent of the differences observed between mental health constructs, use-frequencies and platforms would be valuable future research.

In summary, our results amplify the importance of attending to complexity when measuring and analysing social media use and mental health and well-being. It is important to note that our results do not, and cannot, imply that different types of social media use cause poorer or better health outcomes in young people, but they do provide vital contextual information on user groups that can help us better understand the reasons that previous research has found conflicting results. We have provided estimates of seven well-being measures and the prevalence of four key mental health outcomes (depression, disordered eating, suicidal thoughts and self-harm) across the five platforms Facebook, Twitter, Instagram, Snapchat and YouTube, as well as across three use frequencies. Our findings have shown that the demographic and mental health foot-print of each platform is different. Primarily users differ by sex, but when it comes to platforms YouTube is particularly likely to have both male and female users with poorer mental health and well-being across a range of indicators, alongside evidence that daily Instagram users have better overall well-being than daily users of other platforms. Our findings also indicate that relationships between use-frequency and multiple mental health and well-being outcomes are often non-linear, which supports the importance of considering non-linear dose-response relationships between social media and mental health and well-being in future research. Lastly, we saw that the relationship between use-frequencies and well-being changes depending on the measure of well-being used. This means that we cannot conflate different types of well-being, and doing so will likely result in low replicability.

This research has implications for both those who conduct research on the relationship between social media and mental health, and those who study mental health prediction. We must ensure we are considering both platform-specific and outcome-specific effects rather than conflating types of social media use, social media sites and well-being as single entities. Future research should also stratify results by sex since it is unlikely that studies with differently balanced samples will replicate. Our findings on use-frequencies also suggest that we cannot assume linear relationships between social media use and mental health. Our understanding of these methodological issues would be improved by examining profiles of different user age-groups, as well as examining relationships between these variables longitudinally to understand the potential for reciprocal effects. The differences between platforms should be further considered too, as to how different content types and communication modes on different platforms may affect mental health differently.

## Supplementary Material

Supplementary information The online version contains supplementary material available at https://doi.org/10.1038/s44184-022-00011-w.

S1

## Figures and Tables

**Table 1:** The number of participants in each of the two samples used in this study, subset by demographic characteristics.

Characteristic	ALSPAC cohort	Main sample	Sub-sample
	*N* = 14,901	*N* = 4083	*N* = 2991
*Sex*			
Female	49%	66%	69%
Male	51%	34%	31%
Missing (*N*)	23		
*Ethnicity*			
Ethnic Minority Groups	5.0%	3.7%	3.5%
White	95%	96%	97%
Missing (*N*)	2829	461	332
*A Levels*			
No	23%	19%	19%
Yes	77%	81%	81%
Missing (*N*)	10,801	1384	786
*Parental Employment Class* ^a^			
Non-manual	68%	76%	77%
Manual	32%	24%	23%
Missing (*N*)	3406	566	407

a Parental employment was collected pre-birth of participants.

**Table 2:** The percentage of each demographic group by their self-reported frequency of using any social media each day.

Characteristic	Percentage of group using social media at each frequency			*p*-value^b^
	More than 10 times a day *N* = 1576 (39%)^a^	2–10 times a day *N* = 2144 (53%)^a^	Once a day or less *N* = 356 (8.7%)^a^	
*Sex*				<0.001
Female	40	53	7.1	
Male	35	53	12	
*Ethnicity*				0.4
Ethnic Minority Groups	35	58	6.8	
White	39	52	9.0	
Unknown	41	52	7.2	
*A Levels*				0.3
No	38	52	9.9	
Yes	38	54	7.9	
Unknown	39	51	9.6	
*Parental Employment Class*				0.5
Non-manual	38	53	9.1	
Manual	41	51	8.0	
Unknown	39	52	8.3	

a %.

b Pearson’s chi-squared test.

**Table 3:** The percentage of each demographic group who indicated that they had an account on each of the social media platforms considered.

Characteristic	Percentage of group using each platform				
	Facebook *N* = 3977 (97%)^a^	Twitter *N* = 2294 (56%)^a^	Instagram *N* = 2803 (69%)^a^	Snapchat *N* = 2864 (70%)^a^	YouTube *N* = 2989 (73%)^a^
*Sex*					
Female	98	56	76	73	68
Male	97	57	54	64	83
*Ethnicity*					
Ethnic Minority Groups	95	58	68	73	74
White	98	57	68	70	73
Unknown	96	52	70	72	73
*A Levels*					
No	98	51	68	72	70
Yes	98	58	68	70	73
Unknown	97	55	71	70	74
*Parental Employment Class*					
Non-manual	98	57	68	69	73
Manual	98	55	71	72	73
Unknown	96	54	70	72	73

a %.

## Data Availability

The datasets analysed during the current study are not publicly available as the informed consent obtained from ALSPAC participants does not allow data to be made freely available through any third party maintained public repository. However, data used for this submission can be made available on request to the ALSPAC Executive, with reference to project number B3227. The ALSPAC data management plan describes in detail the policy regarding data sharing, which is through a system of managed open access. Full instructions for applying for data access can be found here: http://www.bristol.ac.uk/alspac/researchers/access/. The ALSPAC study website contains details of all the data that are available (http://www.bristol.ac.uk/alspac/researchers/our-data/).

## Code Availability

The code used to produce the results in this study can be found at https://doi.org/10.17605/OSF.IO/RKXM6.
