# Supplementary material for: The mental health and well-being profile of young adults using social media
Source: Npj Ment Health Res. Author manuscript; Available in PMC 2023 Nov 22. (PMC7615321; doi:10.1038/s44184-022-00011-w)
Supplement: S1 [file EMS186639-supplement-S1.pdf]

Supplementary materials for *The mental health and well-being profile of young adults using social media*

Supplementary Table 1: Percentage of the users of each social media site by use-frequency and demographics reported.

		**Sex**		**Ethnicity**		**A Levels**		**Parental Employment Class**	
Platform	Frequency	Female	Male	Minority Ethnic Groups	White	No A Levels	A Levels	Non-Manual	Manual
Facebook	Daily	90.6	81.8	86.5	87.5	86.7	88.7	87.4	88.8
	Less	7.0	15.2	9.0	10.2	10.8	8.9	10.1	8.9
	Never	2.4	3.0	4.5	2.3	2.5	2.3	2.5	2.3
Twitter	Daily	13.4	20.8	9.8	16.5	16.1	15.6	15.9	17.5
	Less	42.5	36.4	48.5	40.3	35.6	42.6	41.4	37.7
	Never	44.1	42.8	41.7	43.2	48.3	41.8	42.7	44.9
Instagram	Daily	57.9	31.0	53.8	48.7	43.6	49.3	48.7	49.3
	Less	18.4	23.2	15.2	19.9	24.2	18.4	19.1	21.7
	Never	23.7	45.8	31.1	31.4	32.2	32.3	32.2	29.0
Snapchat	Daily	39.9	28.8	44.7	35.9	40.6	33.9	33.9	43.0
	Less	33.6	35.2	28.8	34.1	31.3	36.2	35.3	29.4
	Never	26.5	36.0	26.5	30.0	28.1	29.9	30.8	27.5
YouTube	Daily	22.5	48.4	34.1	31.6	29.2	30.8	31.1	34.2
	Less	45.8	34.9	40.9	41.8	41.2	42.8	42.5	39.3
	Never	31.7	16.7	25.0	26.6	29.6	26.4	26.5	26.5

Supplementary Table 2: The percentage of the sample who had experienced each of the four categorical outcomes.

	Percentages by Sex	
Characteristic	% Female (CI)[1]	% Male (CI)[1]
Depression*	22 (20, 24)	16 (14, 18)
Disordered Eating	10 (9.3, 12)	2.5 (1.7, 3.5)
Suicidal Thoughts	18 (17, 20)	17 (15, 19)
Self-Harm	9.7 (8.7, 11)	4.8 (3.7, 6.1)

[1]CI = Confidence Interval

*Depression was measured in the sub-sample (N=2,991)

Supplementary Table 3: Summary statistics for well-being outcomes, all measured in the sub-sampled (N=2,991).

	Mean (SD) by Sex			
Characteristic	Female	Male	Min. Value	Max. Value
BPN (Autonomy)[1]	5.12 (0.92)	4.99 (0.89)	1.00	7.00
BPN (Competence)[1]	5.03 (1.06)	4.98 (1.04)	1.00	7.00
BPN (Relatedness)[1]	5.68 (0.91)	5.47 (0.90)	1.25	7.00
Satisfaction With Life	23.97 (6.60)	23.15 (6.72)	5	35
MIL (Presence)[2]	23.60 (6.54)	22.36 (6.89)	5	35
MIL (Search)[2]	19.88 (7.07)	20.23 (7.15)	5	35
Life Orientation Test	13.30 (4.59)	14.16 (4.51)	0.0	24.0
WEMWBS[2]	48.33 (8.87)	49.67 (8.97)	14	70
Gratitude Questionnaire	35.15 (5.72)	33.47 (5.79)	7.0	42.0
Subjective Happiness	4.86 (1.26)	4.82 (1.31)	1.00	7.00

[1]Basic Psychological Needs (BPN)

[2]Meaning In Life (MIL)

[2]Warwick Edinburgh Mental Well-being Scale (WEMWBS)

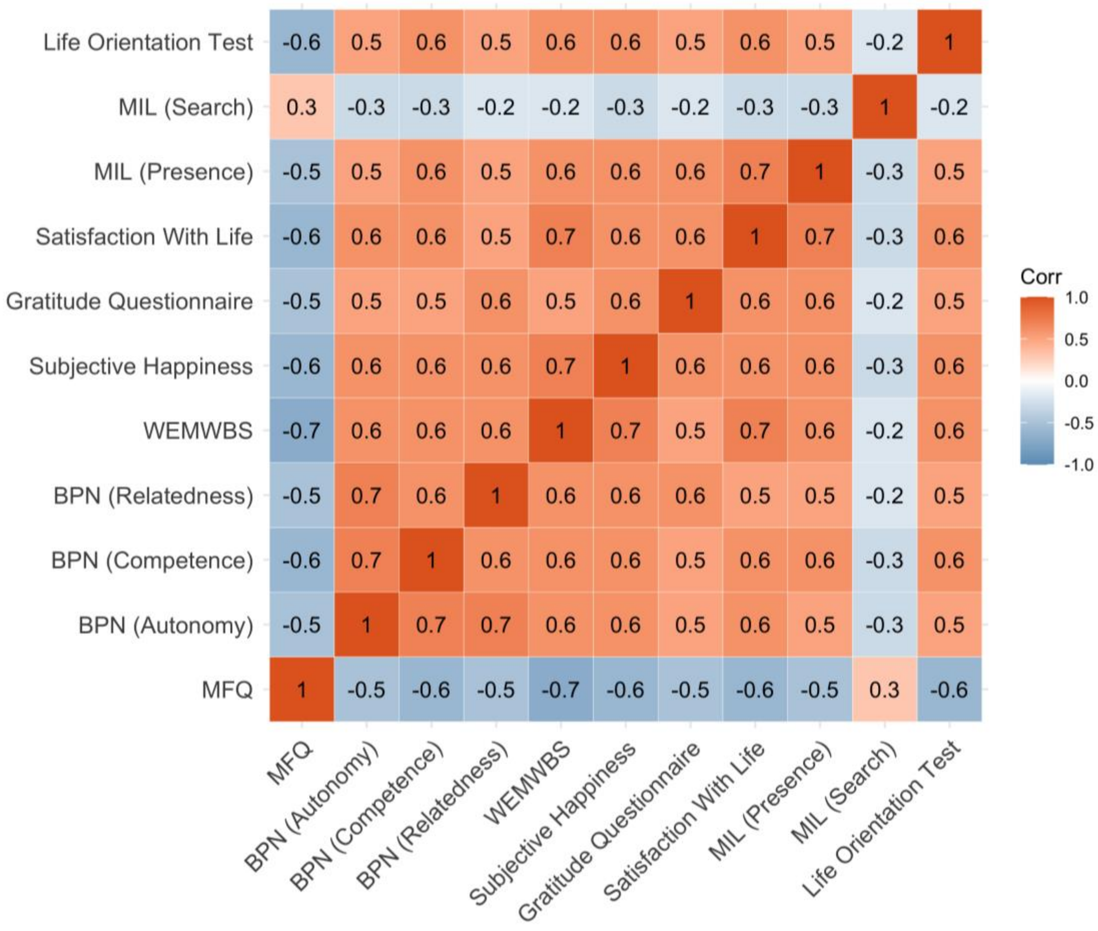

Supplementary Figure 1: A correlation matrix for all continuous mental health and well-being variables using Spearman's Rank coefficient (all $p < 0.000$).

Supplementary Table 4: Contingency table of suicidiality and disordered eating (N=4,083).

	Disordered Eating		
Characteristic	No	Yes	Total
**Suicidality**			
No	77%	5.4%	82%
Yes	15%	2.3%	18%
**Total**	92%	7.7%	100%

Supplementary Table 5: Contingency table of suicidiality and self-harm (N=4,083).

	Self-harm		
Characteristic	No	Yes	Total
**Suicidality**			
No	79%	2.9%	82%
Yes	13%	5.2%	18%
**Total**	92%	8.1%	100%

Supplementary Table 6: Contingency table of disordered eating and self-harm (N=4,083).

	Self-harm		
Characteristic	No	Yes	Total
**Disordered Eating**			
No	86%	6.2%	92%
Yes	5.9%	1.8%	7.7%
**Total**	92%	8.1%	100%

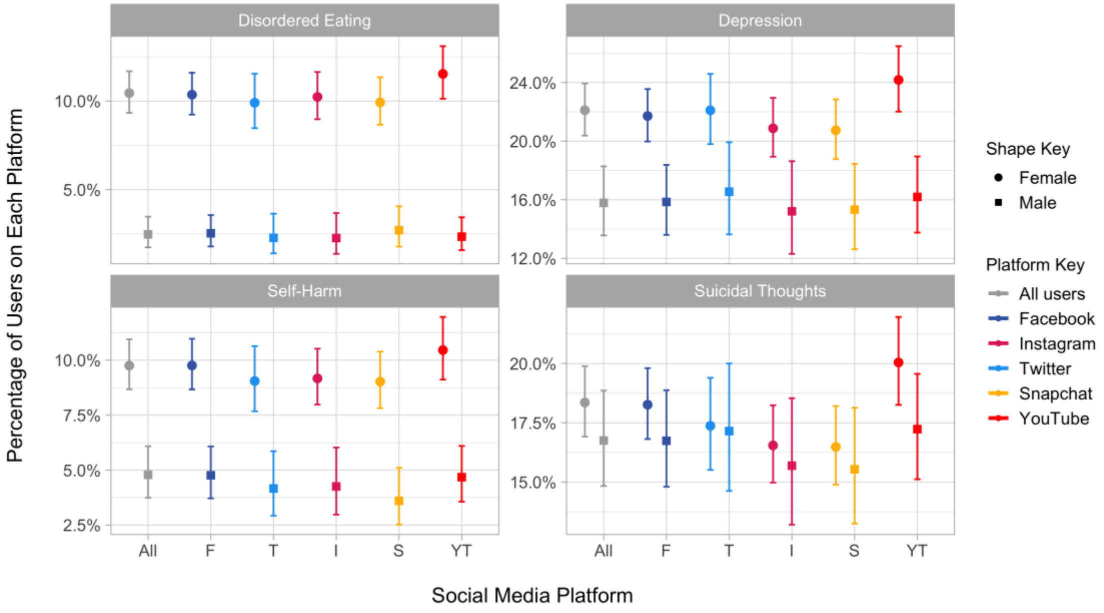

Supplementary Figure 2: Percentage of participants who reported disordered eating, self-harm depression, or suicidal thoughts in the past year, differentiated by sex for all users of each platform, with 95% confidence intervals.

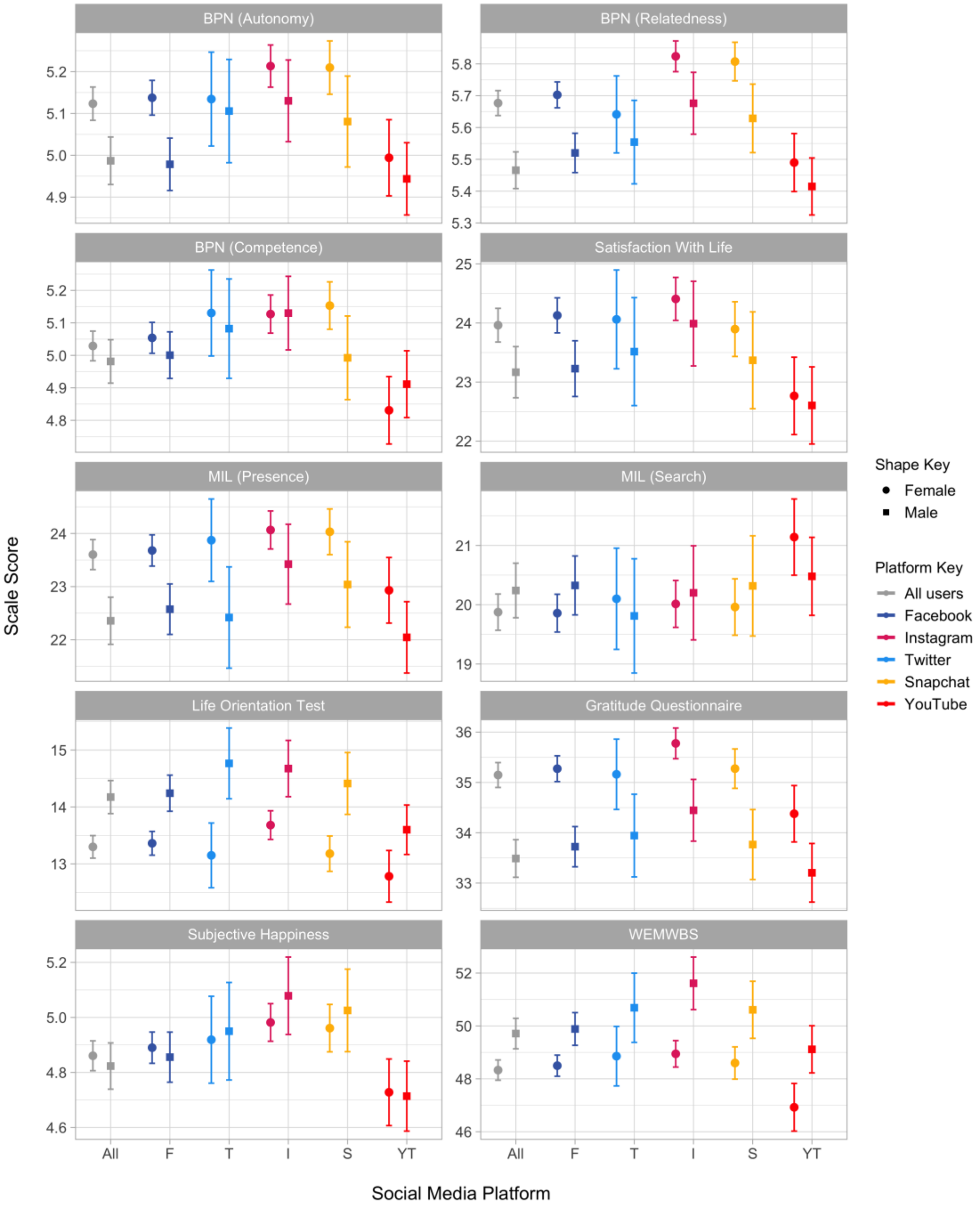

Supplementary Figure 3: Mean scores for seven well-being measures for all users of each platform, stratified by sex, with 95% confidence intervals.
